# Supplementary material for: Homeostatic control of cell wall hydrolysis by the WalRK two-component signaling pathway in Bacillus subtilis
Source: eLife. 2019 Dec 6;8:e52088. doi: 10.7554/eLife.52088 (PMC7299342; doi:10.7554/eLife.52088)
Supplement: Supplementary file 1. [file elife-52088-supp1.docx]

Supplementary File 1. Table of oligonucleotides used in this study

| **Name** | **Sequence** |
| --- | --- |
| oAM473 | GCGCCCGGGGGCAGGAGGAAAATCAAAATG |
| oAM474 | CGCACTAGTTCCCCCATGTAAAAACAAGACTG |
| oAM475 | CTTTTCCGACTGAGCTGAAAG |
| oAM476 | CTGAGCGAGGGAGCAGAATTATCCATCATTTTGATTTTCCTC |
| oAM477 | GTTGACCAGTGCTCCCTGTATAAAGAGGAACAAGAGGATG |
| oAM478 | ATGACTTCATATTCTTTGCCGG |
| oGD59 | CGATGATAAGCTGTCAAACATGAGAATTCCCAGATAAGCTTACATAAGGAGGAACTACTATGAGTAAAGGAGAAGAACTTTTCAC |
| oGD60 | GGTAATGGTAGCGACCGGCGCTCAGGATCCTTATTTGTATAGTTCATCCATGCC |
| oGD68 | GATAAGCTGTCAAACATGAGAATTCGGCGTATTTTTTGTGAG |
| oGD69 | GTAGTTCCTCCTTATGTAAGCTTTCAAAATCTCTTTCTCTTATG |
| oGD137 | CGAGAGAGCTTGAAGATGC |
| oGD148 | GTAGTTCCTCCTTATGTAAGCTTAGCTTTTTATTGGCGGCTAAC |
| oGD149 | GATAAGCTGTCAAACATGAGAATTCGAAATCCTTCATGTAAAGGAAC |
| oGD150 | CTGAGCGAGGGAGCAGAAGGAGTAAGTAATATCGTTTTTATATTTTCACGCTTCAT |
| oGD151 | GTTGACCAGTGCTCCCTGGTCCAATGAAAATAATGGAGTGAG |
| oGD152 | CTTATAGATGACGCCAGAGC |
| oGD165 | GGCTTTGACAGCAGCGGATTC |
| oGD166 | GAATCCGCTGCTGTCAAAGCC |
| oGD172 | CAGAAAAGAGGCGGCCTGTATGGCCGAATTCCCGTCCCGAGCCACTTACC |
| oGD173 | CCATGCTAGCATCTCGAGACACTAGTCAAGACTGATGACGACGAGTACC |
| oGD188 | GGAGCTTGCGAAAGGGAATTTCTCGAG |
| oGD189 | GGGATCCATGCTAGCATCTCGAGCGCTAAATTGCAAGCTCATG |
| oGD214 | GGTAGAGAAGTCGCTGATCAGCGCGAGTATGGAAGATGTC |
| oGD215 | GCTGATCAGCGACTTCTCTACC |
| oGD227 | GAATGAAGCTTGAGCTAGCATCTCGAGACATAAGGAGGAACTACTATGAAGCGTGAAAATATAAAAACG |
| oGD228 | ACCTCAAATGGTTCGCTGGGATCCTTATTCCACTCCACTGTTAG |
| oGD229 | CCTCAAATGGTTCGCTGGGATCCTTATACCGTTTCAGACAGCTTTTGGG |
| oGD278 | GCGCTCTAGAGAATAAGGTTGGTTTTTTTCGGTCGATCC |
| oGD279 | CGCGGTACCTCACGCTTCATCCCAATCATCCTC |
| oGD282 | GCGTCTAGAGAAGCGTGAAAATATAAAAACG |
| oGD283 | CGCGGTACCTTATTCCACTCCACTGTTAGC |
| oGD284 | GCGCTCTAGAGGAGTGGAATAAGACAAAATC |
| oGD285 | CGCGGTACCTCATTGATCTGTATCTAAAATCG |
| oJM104 | CCACCGAATTAGCTTGCATGCAATGAACGGGTTTTCTCTAAAAATTAG |
| oJM117 | CTTCCTGCAGTCACCCGGGCTTATTGAACAACACGTCTTACAACAC |
| oJM320 | CTCACAGAGAACAGATTGGTGGTGGCACTGTTATCAGCAACTC |
| oJM342 | TCGGTGAAAATTGGAAGGCAG |
| oJM343 | CTGAGCGAGGGAGCAGAATTCACCTCAGTTCCTCCCTATAAC |
| oJM344 | GTTGACCAGTGCTCCCTGTAAAGGGGTGCTGCACTCATG |
| oJM345 | ACATTCTATAGTCTGCGCCAC |
| oWX438 | GACCAGGGAGCACTGGTCAAC |
| oWX439 | TCCTTCTGCTCCCTCGCTCAG |
| oYB54 | CGCCTCGAGTTAGAATCTTTTCGCACCGAGGTAAC |
| oYB66 | GCGGGATCCCAAGTATTGAAGGTGAAAGGAACAAGC |
| oYB178 | GGAATGTATTCCGGAAACAAAGTCG |
| oYB179 | CTGAGCGAGGGAGCAGAAGGATCCCTCTTCTCATGAGTGCAGC |
| oYB180 | GTTGACCAGTGCTCCCTGGTCCCCGGCGAATAAAGACCTGAATTTATTTAG |
| oYB181 | GAAGAATAGCTTTTAAGCGTCCGC |
| oYB189 | CAATTTTGAGGGTTGCCAGAGTTAAAGGATCCTGCTAGCTTATTTTTGACACC |
| oYB190 | GATGTCACAAGCAGCTGGGAAGGAATTCTTGAAAACCTGCATAGGAG |
| oYB195 | CTGAAGCTTAACTCTATATATATGTATCTCTTTTTTTAAATTAATCTTATTGATTTC |
| oYB196 | CCTGAATTCGTTGAAAAACATCCCATAAAACATGAC |
| oYB213 | TGGACGGCCATCATCCCTAC |
| oYB214 | CTGAGCGAGGGAGCAGAAGGAAGCACTGGTTTCACCTCAGTTC |
| oYB215 | GTTGACCAGTGCTCCCTGGTCATTGAGAGAAACAGAAAATATATTATTGCGATGTG |
| oYB216 | CTGTAAAGTACGTCCCGACTCC |
| oYB245 | AATTAAGCTTGTCCCGGGTAAACATAAGGAGGAACTACTATGAAAAAGCAAATCATTACAGCTACGAC |
| oYB246 | GAATCCTTTCGCCAGACCAGTACCTGATGTAGATGACGTTTTGCTGC |
| oYB247 | CATCTACATCAGGTACTGGTCTGGCGAAAGG |
| oYB248 | CCACCGAATTAGCTTGCATGCTTAATCAAACCGTAGCTGCGGC |
| oYB257 | GAATTAGCTTGCATGCGGCTAGCCCTGATGTAGATGACGTTTTGCTGCTTG |
| oYB260 | GCAGCAAAACGTCATCTACATCAGGGTCAGGCAGCGCAGAAGAAGCAGGTG |
| oYB261 | CCACCGAATTAGCTTGCATGCGGTTATTGGAAGTATTTCTCTAATCCGCTTTC |
| oYB262 | GCAGCAAAACGTCATCTACATCAGGGTCAGGTGGAAGGCGGATTCATG |
| oYB263 | CCACCGAATTAGCTTGCATGCGGTTACTTCTTTTTTTGGTATTCATATCGCTCC |
| oYB264 | GCAGCAAAACGTCATCTACATCAGGGAAAAAAGGGAATATTGTCACCTATGCAAGAG |
| oYB265 | CCACCGAATTAGCTTGCATGCGGCTAGTTAGGAATCATCTCCAAGTGGG |
| oYB266 | GCAGCAAAACGTCATCTACATCAGGGCAAAAGCTGGTCATTTCCGGAAAAG |
| oYB267 | CCACCGAATTAGCTTGCATGCGGTTAGAAATATCGTTTTGCACCGAGATAGC |
| oYB268 | GCAGCAAAACGTCATCTACATCAGGGTCAGGGTCAAACATTCAAATAGGTTCGAAG |
| oYB269 | CCACCGAATTAGCTTGCATGCGGTTAAAAATAACTTCTTGCGCCCAAGTAC |
| oYB270 | GCAGCAAAACGTCATCTACATCAGGGTCAGGCAAAGGCGTACGTTATCGTCTGG |
| oYB271 | CCACCGAATTAGCTTGCATGCGGTTAGCTGCGGCTGAGAACC |
| oYB288 | GCAGCAAAACGTCATCTACATCAGGGGGTACTGGTCTGGCGAAAGG |
| oYB289 | CCACCGAATTAGCTTGCATGCGGTTAATCAAACCGTAGCTGCGGC |
| oYB336 | ATAGATTCCCATCAGAAATAGTCTACTGTTCATGTGACAAATTTCTTAATTATTCGTTCT |
| oYB337 | GTCACATGAACAGTAGACTATTTCTGATGGGAATCTATCCTTATAATAGAAATCAATAAG |
| oYB351 | ﻿TGAGGGTTGCCAGAGTTAAAGGATCCATCTCGAGGCAAGCTCATGTTTG |
| oYB352 | CCCTTCCCGCTCTTGATGTCGAATTCGCCTGTCGCTAATGCTGTTCC |
| oYB353 | GAAAATCGCCATTCGCCAGGGGGATCCGCAATGAATACGGAAACAGAAATGGCG |
| oYB359 | ﻿GCCAGAGTTAAAGGATCCATCTCGAGTCAGCCTTCATACGTGATATGATCGG |
| oYB361 | ﻿AATAAAATGCATCTGTATTTGAATGAAGCTTACATAAGGAGGAACTACTATGGAGTGGAATAAGACAAAATCAATCTTC |
| oYB376 | TTGCCGATGATAAGCTGTCAAACATGAGAATTCGTTGAAAAACATCCCATAAAACATGAC |
| oYB377 | ATAGTAGTTCCTCCTTATGTAAGCTTAACTCTATATATATGTATCTCTTTTTTTAAATTAATCTTATTGATTTC |
| oYB398 | ATTAAGCTTACATAAGGAGGAACTACTATGAAAAAATGTCTTCTATTTCTAACAACCATTGC |
| oYB399 | AGTTACTAGTCCGCTTATACCGGGCG |
